# Report on the Diseases of Children

**Published:** 1859-01

**Authors:** D. Francis Condie


### REPORT ON THE DISEASES OF CHILDREN.

By D. Francis Condie, M.D.

*Cerebro-spinal Meningitis.*—There would appear to be a propriety in including this affection among the diseases of children, from the fact that, in a very large proportion of the cases which occur, whether in the endemic or sporadic form of the disease, its subjects are children at or under the age of puberty.

During the two past years the disease appears to have prevailed very extensively as an epidemic in different portions of the United States, but more especially in the northwestern portions of the State of New York.

*Description of the Disease.*—The following is the description of the disease, as given by Dr. J. V. Kendall, of Clay, Onondaga County, New York, (*Trans. Med. Society of the State of New York for the year* 1858,) derived from personal observations made by him during an epidemic of cerebro-spinal meningitis, which occurred in his vicinity during the winter of 1857.

There were in a large number of cases premonitory symptoms of some hours' duration, a sense of languor and restlessness, but often scarcely to an extent to attract attention. These premonitory symptoms were succeeded by a chill, not severe, but of several hours' duration, and accompanied or followed by violent pain of the head, usually described by the patient as extending completely through the head from temple to occiputs. A violent pain also generally extended down the neck, or the whole length of the spine, in some cases even over the entire surface of the body. After a time, perhaps some hours from the onset of pain in the head, retching and vomiting set in, and in many cases were frequent and distressing, but in others they were only of occasional occurrence.

After a short period, commonly from twelve to forty-eight hours, the foregoing symptoms abate, the patients feel easier, and there is frequently a softening of the surface, and a general state of improvement, as though the fever had ceased and a speedy convalescence was about to occur. And such was the case in many instances, but, in the majority, there speedily ensued a state of disease to be described hereafter.

In conjunction with the pain in the head, vomiting, etc., there was usually considerable mental disturbance. Occasionally the patients exhibited merely slight confusion of intellect, talking, perhaps, at random, but answering rationally questions put to them, but with apparent effort, and only in monosyllables. Other patients were wildly excited, raving and talking, and tossing about upon the bed, so as to require the aid, sometimes, of one or more assistants to restrain them. Others, again, would lie in a torpid state, with their head drawn backward upon the spine, giving no reply to questions addressed to them; apparently either not hearing or understanding them; while others, again, would manifest a vague comprehension of the questions put, or directions given to them, but seemingly without mental or physical strength to answer or comply. In these latter cases the eyes are closed, or if opened, have a dim, unmeaning look. In nearly all the cases the pupil was moderately dilated,—in a few, however, it was slightly contracted, or of its natural size.

As early sometimes as the second day, but later in the milder cases, petechiæ made their appearance, first on the face, neck, and breast, and extending, more or less, over the entire surface, varying in size from a mere point to several lines in diameter, and in color, from a scarlet to a dark purple, almost black. In some cases the petechiæ were few in number and scattered, in others they covered one-fourth of the entire surface of the body. In the first case, being most generally of a scarlet or rose color, and in the latter, larger in size and of a darker hue. In favorable cases the petechiæ remained but a few days, gradually fading, and, in a week, disappearing. If the patient continued to sink, the petechiæ remained, and grew constantly darker until death. In a majority, probably, of the cases, no petechiæ could be detected by the eye,—in even some of the fatal cases they were absent.

The secretion of urine is moderate in quantity; it is apt to accumulate in the bladder, and to require the catheter for its evacuation. It is commonly dark colored, and muddy, letting fall a thick sediment after standing awhile; sometimes it is as dark as brandy, and without sediment. In the course of the disease it may become clear and of a lighter color, and again, after a few days, assume a muddy appearance.

The bowels are remarkably torpid, resisting the action of large doses of active purgatives.

In the early stage of the disease the tongue was coated with a thin whitish, or a thicker yellowish fur. It was sometimes red and dry—more generally moist. In the advanced stage it is occasionally dry, but more often moist and red. In many cases it would remain for many days moist and apparently healthy.

In cases which continue beyond the fourth or seventh day, the bowels continue torpid. In a very few cases, however, a slight diarrhœa occurred.

There was present a peculiarity of pulse which is more easily recognized by the touch than described. In the more violent cases, soon after the attack, the pulse was feeble, fluttering, easily compressed, often intermittent, and generally very frequent. The intermission of the pulse was most decided in cases attended with a slow pulse—from thirty-two to fifty in the minute. As a general rule the pulse was frequent throughout every stage of the disease—being seldom less than one hundred in the minute, and occasionally running up to a number scarcely to be counted. It varied much, however, from day to day, without any perceptible cause.

As in the commencement of the attack, so also in the latter stages of the disease, there was frequent retching, with, occasionally, distressing vomiting; ejecting, first, such substances as have been taken into the stomach, and afterwards its viscid and acid secretions.

DR. T. H. SQUIRE presents, in the same work from which we derive the foregoing, the history of forty-three cases of cerebro-spinal meningitis, with the view to its faithful delineation in the most fatal form under which it prevailed, during the winter and spring of 1857, in Elmira and other villages of Chemung County, New York.

The symptoms, he remarks, were very uniform in all the cases. A chill, headache, vomiting, prostration, morbid sensitiveness of the skin, jactitation, coldness of the surface, wildness of expression, dilated pupils, irregular breathing, paralyzed deglutition, wry neck, retraction of the head, dullness or abolition of the senses, pulse but little affected, bowels quiescent, petechiæ, delirium, convulsions, coma. Many or all of these symptoms are present in every case. The diagnosis is therefore not difficult, especially when the disease is fully established.

*Subjects of the Disease.*—Of the forty-three cases reported by Dr. Squire, thirty-one were in males, and twelve in females. Seven of the patients were between 1 and 2 years of age, seven between 4 and 5 years, eight between 5 and 10 years, five between 10 and 15 years, five between 15 and 20 years, four between 20 and 25 years, and seven between 30 and 41 years. Thus, the same nearly as in all other epidemics of cerebro-spinal meningitis, children and young persons, especially boys, were by far the most frequent victims of the disease.

*Duration.*—In regard to the duration of the disease in the forty-three cases recorded by Dr. Squire, 2 terminated in two hours, 2 in four, 3 in six, 1 in seven, 4 in twelve, and 1 in sixteen hours; 5 terminated in one day, 5 in two, 6 in three, 3 in four, 4 in five, 1 in six, 1 in nine, and 1 in ten days; in two cases the disease continued for eight weeks, in one four months, and in one ten months.

*Further Description of the Disease.*—From a very interesting report on cerebro-spinal meningitis as it occurred epidemically in Churchville, New York, in the latter part of the winter of 1857–58, and in the spring of the latter year, communicated to the *Buffalo Medical Journal* for July, 1858, by Dr. J. W. CRAIG, we copy the following description of the disease.

It seemed to have assumed two distinct forms, namely, a mild and a severe one; or what might, perhaps, have been denominated cerebro-spinal typhus, mitior and gravior.

"The mild cases were ushered in with a chill, more or less severe, followed by fever, pain in the head, extending down the neck and back, and accompanied with a feeling of general uneasiness. This set of symptoms generally continued from eight to twelve hours, when an intermission occurred, more or less complete; but about the same time next day there was a return of the pain and fever, but no chill. The pain and fever continued to occur from one day to another; the patient, during the intermissions, being, in most cases, able to move around the house.

"The more malignant cases were also ushered in with a chill, followed by headache, vomiting, double vision, strabismus, intolerance of light and sound, with deafness and swollen tongue; convulsions in children, and delirium in adults. In some cases the skin presented a mottled appearance, with spots like *taches rouges;* in other cases there were petechiæ, with cold extremities, feeble pulse, and ultimately death. When reaction did take place—which had necessarily to be speedy, as these cases terminated often in from four to twentyfour hours from the accession of the chill, the extremities became warm, the pulse fuller, the delirium less constant, and slight febrile reaction manifested itself. In the most favorable cases of this severer form of the disease, after reaction became fully established, the pain in the neck and head continued; the head was drawn stiffly backward, the same rigidity extending down the whole length of the back, and to such an extent that to raise the lower extremities was to raise the whole body. The morbid sensibility to light and sound continued, as also the dilated pupil and strabismus, and, in many cases, at particular hours, there were convulsions, and a *projectile* vomiting of a green or yellow matter. In addition to the foregoing symptoms, there were, also, great restlessness, jactitation, pain in the stomach and bowels, with anorexia, which continued for a number of days."

A marked peculiarity of many cases of the disease was, the unexpectedly sudden occurrence of the attack, and its terrible severity. The patient may retire to bed apparently in his usual health, and soon after falling asleep be awoke by a chill, and the family aroused by unmistakable symptoms of convulsions. In other cases, after eating a hearty dinner, the patient will be taken with a chill, soon followed by delirium, and this without any premonition whatever. Sometimes a sense of uneasiness was experienced, for twenty-four or forty-eight hours previous to the attack, but in the majority of the severe cases, the prodromic symptoms are so slight as to escape notice.

The delirium in this disease is peculiar; in many cases it had several of the characteristic features of delirium tremens. "In children there is a strong tendency to pulling their hair, biting their lips, fingers, etc., so that they require constant watching to prevent them from injuring themselves. They will also start from their beds suddenly as if fearful of approaching danger."

*The Post-mortem Appearances* in six cases which proved fatal in less than forty-eight hours from the attack, were as follows:—

"In all of them, on removing the calvarium, there was observed a turgid condition of the venous system. On removing the coverings of the brain, lymph of a yellow and greenish hue was detected in the upper sulci of that organ; and in all there were increased quantities of lymph with sero-purulent effusion at the base of the brain, and extending down the whole length of the spinal cord. The choroid plexus was also very much engorged, and more or less serous effusion in the ventricles. We also found softening of the base of the brain and upper portion of the spinal cord; more marked, however, in some cases than in others."

Two cases, which proved fatal several months after the attack, were examined. Both were of the severe form of the disease; convalescence was very slow. Still the patients seemed to rally, improve in strength, and gain flesh for several weeks, so that they were able to do considerable work. After they had become able to be about the house, they would be attacked every week or ten days with a sense of faintness, headache, and vomiting, which would last for from one to six hours, and then disappear, leaving the patient apparently as well as before their accession. In the forenoon, on one occasion, the patient, a female, had drawn a child, in a hand-wagon, for about a mile; in the course of the day she was attacked with the most formidable symptoms of the disease—dilated pupils, strabismus, double vision, vomiting, headache, intermittent pulse, etc. Every five minutes breathing would seem to become suspended, the patient continuing still perfectly conscious and rational. Death finally occurred, apparently from paralysis of the respiratory muscles. The symptoms of the other case were very nearly the same.

In both cases a post-mortem examination revealed a softened condition of the base of the brain and upper portion of the spinal cord, with a very copious effusion of limpid fluid in the ventricles; amounting in the one case to eight, and in the other to twelve ounces. It should have been mentioned that in both cases there was paralysis of the scalp on the back part of the head, and of the muscles of the back of the neck, with complete numbness.

In 129 cases that fell under the notice of Dr. Craig, twelve only proved fatal. Of these twelve cases, in five death took place within thirty-six hours; in three within seven days; in one not until the fifth week, and in another not until the eighth. In two cases death occurred not until several months from the onset of the disease. The mean duration of the disease was about three weeks.

Continued pain of the head, stiff neck, quick pulse, delirium and coma, were unfavorable symptoms; occasionally by good management, a favorable termination was obtained, under circumstances the most forbidding.

*Further Description of the Disease.*—In the Report of the Beaver County Medical Society, (*Trans. of the Medical Society of the State of Pennsylvania, New Series, Part II.*, May, 1857,) DR. S. CUNNINGHAM states, that "Epidemic cerebro-spinal meningitis prevailed in the spring of 1848, in a very malignant form. The attacks were mostly sudden. The patient would be seized with violent pain in the head, most severe in the occiput, but frequently felt in the temples, and over one or both eyes; the pupils of the eyes were mostly contracted, but sometimes dilated; great intolerance of light; the brows knit; throbbing of the carotids; severe pain in the back of the neck and along the spine; in many cases violent opisthotonos; during the paroxysm the body would be raised entirely from the bed, the head and heels alone touching; delirium and coma in the advanced stages; obstinate constipation, it being with great difficulty that an evacuation from the bowels could be procured; the functions of the kidneys were partially or totally suspended. Many cases proved fatal very speedily, while others would linger until the patient had become extremely emaciated; in the latter cases there would frequently be a total loss of sight, and of most of the other senses. We are not aware that any post-mortem examinations were made, the people in the country being greatly opposed to anything of the kind."

*Etiology.*—In regard to the etiology of cerebro-spinal meningitis, Dr. Kendall (*op. citat*, page 108,) remarks, that "To attempt to delineate the causes of this fearful disease would be as difficult as in respect to any other epidemic disease; or to explain why it should prevail in my locality, and leave untouched the village and vicinity of Baldwinsville, but three miles to the westward, situated upon the same river, and with apparently the same causes existing to produce it, would be equally difficult. It is proper, however, to remark, with respect to a general cause, that the weather was steadily and excessively cold, and it was observable that as the weather grew colder, new cases of the disease continually occurred, and, as it became milder, they diminished. Thus it fluctuated from time to time, and with the approach of spring the disease nearly vanished."

Dr. CRAIG, in the *Buffalo Medical Journal*, states that in his vicinity the disease was, for the most part, confined to low miasmatic regions; a few cases, however, occurred seemingly outside of these influences; but these were rare, and he thinks that if all the facts were known in respect to them, it would be found that the patients had been exposed to the same exciting causes as those who lived in marshy districts.

The first appearance of the disease was soon after the melting of a large body of snow, by a south wind, in 1857, and "the sudden change in the weather" was the popularly assigned cause of the epidemic; but in 1858, Dr. Craig remarks, no such cause could be given. He believes it can be shown that, in his neighborhood at least, the disease was confined to miasmatic localities. During the epidemic there was a prevalence of miasmatic diseases—intermittents and remittents—of unusual persistence and severity; this, with the marked periodicity in the phenomena of the milder cases, and of the latter stages of the more severe, Dr. Craig believes to show conclusively that the disease was of miasmatic origin.

*Treatment.*—In respect to the treatment of cerebro-spinal meningitis, Dr. Kendall remarks that venesection was practiced in one case with a favorable result, but in no other dared he resort to the remedy, on account of the evident tendency, which was invariably present, to rapid and fatal prostration. Anodynes, even in large doses, seemed in the early stage to increase the watchfulness; but subsequently, after an evacuation from the bowels had been procured, they often quelled the intense restlessness of the disease like a charm. In some cases, cold applications to the head appeared to be pleasing to the patient; in other, and the majority of instances, warm fomentations, as of hops steeped in warm water, applied to the scalp, with heat to the lower extremities, were productive of a good effect.

In cases in which the brain and spinal nerves had become, as it were, benumbed much immediate benefit was derived from the application of several blisters to various parts of the surface, particularly upon the neck. When, in the first stage, the patient was semi-delirious, and tossing about with pain, calomel in large doses, was found to be the best remedy—a state of ease and quietness generally ensuing so soon as the calomel produced a free discharge from the bowels. In all stages of the disease, when the bowels are inert, procuring a free discharge of dark offensive stools by the administration of calomel, has the effect of rousing the patient from his state of stupor.

In the early stage of the disease, quinine was found to be utterly useless as a means of arresting its progress. Subsequently, however, to the first stage, when there were still much restlessness and wakefulness, its use, in full doses, sometimes induced perspiration and a state of calmness; still later in the disease, in smaller doses, it had some value as a tonic.

If the patient survive the first period of the attack, and then sinks into a state of indifference, according to Dr. Kendall, nothing is left for the physician to do but merely to watch the progress of the case, and meet whatever symptoms arise with their appropriate remedies. On many days it will be apparent that the efforts of nature are doing as much for the patient without, as they could with, therapeutic aid. Whatever treatment may be necessary during this stage, it must be of an alterative, stimulant, and tonic character.

In the treatment of cerebro-spinal meningitis, Dr. Squire prefers, during the cold stage of the attack, the free use of brandy and other stimulants; but if the patient survive this period, and a severe paroxysm of fever sets in, with unmistakable evidence of cerebral congestion, he questions whether the lancet might not be admissible, or even judicious. It is a sad truth, however, he remarks, that in the more formidable cases all treatment was found to be alike unavailing.

Dr. Craig remarks that in any form of the disease depletion had a mischievous effect. He found that the administration of the mildest cathartic was always followed by an alarming prostration.

"If the patient is seen during the chill, every means should be used to promote speedy reaction, as the warm bath, surrounding the patient with heated bricks, or bottles of hot water; hot drinks, brandy, and ammonia internally; sinapisms to the legs, abdomen, spine, etc. After reaction has been established, the local symptoms are to be combated by cold applications to the head and neck, assiduously persevered in, so as to reduce the violence of the reaction. Should these not afford relief, warm fomentations of hops and vinegar may be substituted; they are often grateful to the patient by quieting pain and procuring sleep. The vomiting may, in general, be relieved by the use of some bitter stomachic infusion—as infusion of quassia, given cold, or by a pill of opium. If these fail, an emetic will often succeed."

In regard to blisters, Dr. Craig has seldom seen any good effects result from them; on the contrary, he has known them to be productive of great irritation and restlessness. Nervous symptoms are to be relieved by the use of valerian, musk, assafœtida, etc. The "sheet-anchor" in these cases, however, Dr. Craig has found to be the sulphate of quinia in doses of from 30 to 40 grains, in the twenty-four hours, as the stomach will bear it. "The subintrant character of the disease in its early stages, will not," he remarks, "allow us to wait for an intermission; but the remedy should be given, in divided doses, every three or four hours, and should the case be prolonged, or of the milder type, I know of nothing more effectual than Fowler's solution, in doses of from three to five drops, three times a day."

*Iodide of Zinc in Chorea.*—We are informed in the *New Orleans Med. News*, that DR. BARLOW still continues to employ the iodide of zinc in the treatment of chorea, when occurring in patients of a strumous diathesis. The remedy was introduced by him some two years ago. In cases of chorea in which there exists no peculiarity of diathesis, Dr. Barlow prefers the sulphate of zinc. Besides the influence which the iodide of zinc exerts over the strumous cachexia in cases of chorea occurring in those in whom such a cachexia exists, it may be useful also in the control of the rheumatic diathesis to which chorea has been shown to be closely related. Good authorities could be adduced who account for the frequency with which affections of the heart are found to complicate chorea, by supposing the latter to be dependent upon a morbid condition of the system very nearly related to rheumatism—resulting from similar causes, and occurring more frequently in individuals predisposed to rheumatic affection than in those in whom no such predisposition exists. A little girl was recently discharged from under Dr. Barlow's care, in whom, under a course of the iodide of zinc, resorted to for the cure of chorea, a loud cardiac *bruit* had become very much diminished in intensity.

*Cholera Infantum.—Etiology.*—DR. ROBERT A. GHOLSON, of Petersburg, Va., in a paper on this disease contained in the *Virginia Medical Journal* for May, July, and August, 1858, denies its dependence on malaria, and endeavors to show that its etiology, as taught by most American authors, who connect it essentially with some peculiarity of our climate in regard to a vitiated atmosphere, intense atmospheric heat, or vicissitudes of temperature, is unsatisfactory and inconsistent with facts. In the first place he maintains it to be an error to suppose cholera infantum to be a disease peculiar to the United States. Neither is the disease, he remarks, confined, as has been asserted, to our large cities, to low unhealthy localities, or to the season of the greatest heat, but in the southern and western sections of our country it prevails alike in town and in country, in the most elevated and salubrious regions, as in low, wet, and malarial neighborhoods, and in spring, fall, and winter, as in midsummer. He therefore maintains, that a disease which would thus seem capable of being developed in almost all climates, seasons, and localities, cannot depend for its occurrence upon atmospherical heat, atmospherical vitiation, or any other atmospherical influence, however much these various conditions of atmosphere may confessedly affect its prevalence and fatality.

It is true, as Dr. Gholson remarks, that there is scarcely a European writer on the diseases of children who does not, under some name or other, describe very accurately cholera infantum, its symptoms during life, and the appearances presented after death. But no European authority, so far as we are aware, has declared the disease to be endemic—widely prevailing and particularly destructive of infantile lives—in the large cities of Europe, during the season of most intense heat. The extent of its prevalence and fatality being always in direct proportion to the heat of the summer and to the crowded and unwholesome character of the entire city or particular sections of it. That such is the case in the United States is proved by statistics, of the entire accuracy of which there cannot be a doubt.

Dr. Gholson insists, and very correctly, that in order to explain the production of cholera in children at the particular age to which its occurrence is almost exclusively confined, and also the universality of its occurrence, we must invoke the agency of those *physiological changes* which dispose so inevitably to certain diseases at certain periods of life: that these changes about the period of primary dentition predispose so powerfully to the occurrence of the morbid phenomena which constitute the disease under consideration, that these become developed under the influence of exciting causes that are almost universal and omnipotent; while the prevalence and malignity of the disease are chiefly influenced by a high atmospherical temperature, especially when associated with atmospherical impurity.

*Nature of the Disease.*—Dr. Gholson presents a brief synopsis of the symptoms, course, etc. of the disease, and of the developments of pathological anatomy, from which he deduces the opinion that it consists essentially in an affection of the follicular and glandular apparatus of the stomach and intestines, attended by a morbidly increased action of their proper functions. That, although the local disease is not necessarily inflammatory in its character, it is nevertheless very prone to run into inflammation in its progress. He considers "the simple uncomplicated *follicular* affection to be the proper *normal* type of the disease, demanding our principal attention in *the great majority of cases*, and which, in all cases, however complicated by other pathological conditions, should never be lost sight of."

Somewhat similar views to those of Dr. Gholson in reference to the etiology and nature of cholera infantum were advanced by DR. EDWARD PARKER, of the City of New York, in a paper contained in the *Transactions of the Medical Society of the State of New York* for 1857. Dr. Parker endeavors to show that, contrary to the generally received opinion of the profession, cholera infantum is not a disease peculiar to this country; that it is not almost entirely confined to large cities; that neither its symptoms, course, nor pathology entitle it to a separate place in our nosological lists; that its treatment is not to be distinct from that of diarrhœa on the one hand, and of dysentery on the other; and that the cerebral symptoms occurring in the advanced stages of the disease are not those of hydrocephalus.

Dr. Parker adduces the descriptions given by Dr. West, of London, of inflammatory diarrhœa, and of Dr. Bouchut, of Paris, of infantile entero-colitis, which, in all their essential features, will certainly represent very accurately a well-marked case of cholera infantum. There can be no doubt that a disease identical with that known among us as cholera infantum does prevail to some extent in different portions of Europe. It is nevertheless true, that infantile cholera is the especial endemic, during the summer season, of the larger cities of our Middle, Southern, and Western States, and that nowhere else does it prevail to the same extent, and is productive of an equal amount of mortality. No dependence is to be placed on the census returns, quoted by Dr. Parker to prove that cholera infantum is equally prevalent beyond as within the limits of large cities. In the year to which they refer, instead of 260 deaths occurring in the entire State of Pennsylvania, as they present, the Reports of the Board of Health of Philadelphia show a total of 505 for that city alone. But it is not denied that the disease may and does occur out of cities. Wherever children, during the period of their first dentition, are exposed to a hot, and at the same time impure, damp, and stagnant atmosphere, and are improperly fed, and are lodged, especially at night, in crowded, filthy, and unventilated apartments, whether in or out of the city, they will be liable to an attack of cholera infantum; but as this concurrence of morbific agents is more frequently met with in the narrow courts, streets, and alleys, and in the badly constructed, illy kept, and over-populated dwellings of the poor and improvident of large cities, it is there that the disease prevails every summer, and is productive of the greatest mortality.

Cholera infantum is by no means a specific disease; it is in its pathological character closely allied to the other intestinal profluvia of infancy. While in its early stage it consists, in probably the majority of cases, simply in an increased and somewhat altered secretion from the mucous follicles and the glandular bodies of the intestines, yet, in its second stage at least, it is, on all hands, admitted to be strictly an entero-colitis. That such is the case is proved beyond the possibility of a doubt by the pathological researches of Dr. Edward Hallowell, as recorded in the *American Journal of the Medical Sciences* for July, 1847.

*Treatment.*—It is evident, therefore, that the general indications of treatment must be the same in cholera infantum as in the inflammatory forms of diarrhœa, and in the entero-colitis of infancy. As the main agents in the production of cholera infantum are unquestionably a high degree of solar heat, a damp, confined, and vitiated atmosphere, improper food, etc., to insure a speedy cure of the disease, it is of primary importance that the patient be exposed to a cool, dry, fresh air, and be furnished with a wholesome, well-regulated diet; with these hygienic measures, and the use of the warm bath daily, but little positive medication will, in the early stage, of most cases be demanded; without them, all other remedies, however well selected or assiduously administered, will very generally fail to produce any permanent good.

In respect to the cerebral symptoms attendant upon the advanced stages of protracted cases, there is no doubt that they are, very generally, the result simply of exhaustion and anæmia, and are totally unconnected with any amount of meningeal inflammation. At a somewhat earlier period of the attack we have, however, in numerous instances, observed the occurrence of all the symptoms of acute meningitis, resulting in early effusion, as shown by repeated examinations after death.

*Etiology.*—In the report of the medical topography of Somerset County, Pa., (*Trans. of the Medical and Surgical Faculty of Maryland* for June, 1856,) DR. HANDY remarks that cholera is much less frequent in that county now than formerly, but to what causes this diminution is to be attributed he has been unable to determine. The disease does not appear to him to depend upon malaria. "It usually commences," he remarks, "in the latter end of May if it is hot, or about the beginning of June; it rarely occurs *de novo*, after the middle of August. I have known it to be present in infants two months old, but have never, to the best of my recollection, seen it after the second summer. There is nothing particular in the disease as it prevails here, except its unusual mildness of form. It is, undoubtedly, the product of excessive heat, more especially when combined with the relaxing effects of moisture, and it is excited by the process of dentition, and imprudence in feeding. As these causes are with difficulty avoided, we always have cases of the disease, though fewer than formerly."

*Morbid Anatomy.*—In the *New York Journal of Medicine* for July, 1858, Dr. J. Lewis Smith presents a report of the post-mortem appearances observed by him in eleven cases of cholera infantum.

In six of these cases the stomach was not examined; in the remaining five it seemed healthy. This fact, Dr. Smith remarks, is the more interesting when we consider that some eminent writers have asserted that the stomach presents the lesions of inflammation, and recollect that one of the prominent and obstinate symptoms of the disease is usually referable to this organ. He finds, however, from his notes, that vomiting was not present, during the period of attendance at the Dispensary, in 46 patients out of an aggregate of 133; and in only 24 of those who suffered from it did it occur at the commencement of the case.

The small intestines appeared natural in three cases; in four they were moderate, or slightly injected in places. In the remaining four, the records state that the jejunum seemed healthy, but the ileum was more or less inflamed; continuously so in only one case. In two cases the ascending colon was not examined; in two it presented the usual healthy appearance; in five it was vascular in patches; and in the remaining two it was uniformly injected. The descending colon was, in all the cases, the principal seat of inflammation, its mucous coat presenting a continued red surface. In four cases, the records state that the mucous membrane was thickened; it may have been so in others. Follicular ulceration was present in five cases. In two or three cases, on tracing the intestine into the pelvis, it was noticed that the signs of inflammation gradually diminished. The liver was inspected in only four of the cases; in three of these there was no appreciable alteration in its condition discovered; in the other case it was congested.

Dr. Smith refers to the following as the important particulars developed by the foregoing dissections :—

"1st. That the stomach, though so irritable in the disease, and the liver, in the few cases in which it was examined, did not present any notable alteration from the healthy state.

"2d. That in all the cases there was well-marked intestinal inflammation. Two died when only five days sick, and yet in both the descending colon presented the inflammatory lesion in a high degree.

"3d. That the inflammation was not confined to the mucous follicles, as it has been stated to be by some writers, but extended in patches over the mucous surface.

"4th. The portion of the intestinal tract in which the inflammation was most intense was, in all the patients, the descending colon, and *colitis* was *the only lesion* invariably present."

*Dysenteric form of Cholera Infantum.*—In the paper of Dr. Gholson, already referred to, (*Virginia Med. Journ.*, May, 1858,) there is described a form of cholera infantum, said to be of frequent occurrence in the Southern and Western States, and attended with great fatality. In this form the intestinal disease is complicated with the prevailing fever of the district in which it occurs. Thus, in the upper portions of Virginia and in some other States, it will be modified by the concurrence of some form of continued fever; but in lower Virginia, and other flat and miasmatic localities, by some form of periodic fever. This form of the disease, especially in the vicinity of Petersburg, assumed a rapid and fatal course. It would seem to prevail often as an epidemic, presenting, in the infant, the analogue of that terrible disease of the adult, "epidemic dysentery." It is generally ushered in by vomiting and purging, attended by a grade of fever and a predominance of nervous symptoms which no existing local affection can possibly explain. In Virginia, the fever, unless from its very onset there occur inflammation of the brain or of the abdominal organs, will either remit or intermit, or exhibit a strong tendency to do so, in the course of twenty-four hours; the stomach and bowels becoming comparatively quiet, and the child emerging from its state of torpor and craving food. In a few hours, however, the feet become cold, the head hot, and the stomach irritable, and there speedily occurs a renewed exacerbation of fever, attended by a state of torpor, from which the patient is aroused by the paroxysms of tormina and tenesmus, with which the greenish, and more or less mucous and often bloody discharges from the bowels are usually accompanied. So far as Dr. Gholson's own observations extend, he has never known cholera infantum to prevail extensively in the country and be attended with much mortality there, without presenting this form.

*Treatment of Cholera Infantum.*—After some judicious remarks on the hygienic treatment of cholera infantum, which correspond, in their general outline, with those of most of the writers on the disease, Dr. Gholson proceeds to consider the remedial treatment. In the first stage of the more simple and milder forms of the disease he trusts its cure to the acetate of lead and calomel. The first of these he considers our most valuable remedy so long as the discharges continue profuse and watery, and no decided indications of inflammation or of prostration present themselves. He gives the calomel alternately with the sugar of lead, in doses of from one-twelfth to one-sixteenth of a grain.

When the discharges are acid, he gives the bi-carbonate of soda in carbonated water; when there is a decided alkaline condition of stomach he resorts to some acid; when there is a tendency to collapse, diffusible stimulants are necessary. To relieve irritability of stomach, his remedies are, the tepid or warm bath, a sinapism to the epigastrium, sinapized pediluvia, stimulant liniments to the spine, and anodyne enemata. In the diarrhœal stage, with mainly undue peristaltic actions of the bowels, nothing, he considers, will be required to insure recovery but a well regulated diet, the tepid bath, and perhaps some cordial laxative, combined with or followed by an anodyne. When the discharges are of a greenish hue, he advises alkalines; when clay-colored or whitish, any of the milder preparations of mercury; when they continue watery and profuse, the acetate of lead. If these means fail, some one of the vegetable astringents, with the addition of a small quantity of paregoric, laudanum, or tincture of hyoscyamus must be given.

When the disease threatens to become chronic, especially if it be attended with much emaciation and debility, Dr. Gholson cautions against the continuance of mercurials, as they often lay the foundation of a state of hopeless cachexia from which no subsequent course of treatment, however judicious and persevering, is calculated to rescue the patient.

When inflammation of the digestive mucous membrane supervenes, but especially when entero-colitis occurs, with decided dysenteric symptoms attended by continued symptomatic fever, Dr. Gholson directs leeches; calomel, alone or combined with ipecac., hyoscyamus, or opium; emollient poultices, followed by warm anodyne fomentations, rubefacients, and blisters. When tormina and tenesmus are severe, he recommends occasional anodyne enemata. When the inflammation is seated higher up, and is still acute, and with no symptoms of prostration, injections of cold water are said to prove beneficial.

In *the inflammatory type* of the disease, the patient should be confined strictly to mucilaginous drinks, with the addition of small quantities of bi-carbonate of soda, or lime water. Ice confined in a thin gauze bag should be given the child to suck. As the acute stage declines there often remains a sub-acute affection of the large intestines, especially the rectum, attended with troublesome tormina and tenesmus. This calls for sedative, astringent, and anodyne injections. The best, according to Dr. Gholson, is a solution of acetate of lead, then the sulphate of zinc and the nitrate of silver. Two or three grains of either of the first two, or half a grain of the last, may be dissolved in two tablespoonfuls of some mucilaginous fluid with the addition of a few drops of laudanum, for an injection, the return of which should be prevented by external pressure.

The disease is sometimes ushered in with great nervous debility and restlessness, hot, swollen, and inflamed gums; a flushed face, and excited eye, and hot head, while the stomach and bowels are, perhaps, comparatively quiet. In such cases, especially if attended with much vascular excitement, Dr. Gholson directs incision of the gums, leeches to the temples, or mastoid processes, cold to the head, revulsives to the extremities, the tepid bath, cathartic doses of calomel, or cathartic enemata. Dr. Gholson is adverse to the use, in these cases, of blisters: in the diseases of children he has found them to be "more frequently *co-irritating* than *counter-irritating* agents." In every case where inflammation of the brain and its meninges is threatened, we should not hesitate promptly to resort to the above remedies; still, in a disease, one of the most striking features of which is rapid emaciation and debility, we are carefully to avoid all unnecessary depletion. Cases may occur attended with starting and indications of alarm at the slightest noise, followed by imperfect sleep, with twitching of the tendons, or even of the muscles of the face, the patient awaking with screams of terror, and perhaps a momentary flushing of the cheeks, and heat of the head, where depletion would do harm—such cases being best controlled by the tepid bath, a composing draught, or anodyne enema, cold to the head, and a dark, cool, quiet room.

Late in the disease a state of drowsiness or stupor often ensues, attended by a piteous wail or moan. The discharges, perhaps, have ceased or notably diminished. In this condition there is no reason to suspect inflammation of the brain; there is, on the contrary, a state of exhaustion—a flagging of the dynamic nervous energies, and nothing but a prompt resort to suitable stimulants will save the child.

In the *epidemic or dysenteric form* of cholera infantum Dr. Gholson recommends the sulphate of quinine, given at the earliest possible period. Any condition of the system incompatible with the quinine, is to be attacked with a boldness and energy that would be inadmissible under other circumstances. So soon as the paroxysm begins to moderate, an enema composed of some two grains of quinine, as many drops of laudanum, mixed in about a tablespoonful, or more, of any mucilaginous fluid, is directed to be given. If this be well borne, the same enema should be repeated every three or four hours. While the quinine is thus given cold may be applied to the head, and poultices, rubefacients, or blisters, to the abdomen So soon as the case is divested of its periodic character, the subsequent treatment is pretty much the same as in the ordinary inflammatory forms of the disease.

*Chronic Cholera Infantum.—Treatment.*—When the disease has become chronic, there is, Dr. Gholson remarks, usually more or less inflammation with ulceration, chiefly of the mucous coat and follicles of the large intestines, attended with much emaciation and debility. Here our chief reliance is on the vegetable astringents; but in cases in which the discharges are mucous, gleety, or purulent, the mineral astringents and acids, and the terebinthinate and balsamic preparations, with an occasional addition of some preparation of opium or hyoscyamus are to be preferred. When the disease is confined chiefly to the lower portion of the large intestine, sedatives and astringents should be administered by the rectum. Dr. Gholson recommends especially the sulphate of zinc, two grains, gradually increased, in one or two tablespoonfuls of some mucilaginous fluid, with the addition of a few drops of water, given two or three times a day. In these chronic cases, the assiduous employment of the warm bath, which may be rendered more stimulating by the addition of mustard or salt; with frictions, rubefacients, and blisters, to the abdomen should not be neglected. When there occur much emaciation and debility, with loss of appetite, tonics and stimulants will be required. When anæmia, with degradation of constitution, is present, Dr. Gholson directs some preparation of iron, as the iodide or citrate, with quinine, etc. Often the best thing we can use, he remarks, is "a good article of old brandy."

In regard to diet no arbitrary rules can be laid down. It will be often found that meat teas, broths, or juices, will agree with the patient far better than milk and the usual farinaceous preparations. There are many cases in which no medicine would seem to do good; here everything depends on sustaining the strength of the child until the occurrence of cold weather. A common practice with the physicians of a part of Virginia, is, we are told, in such cases, to put the patient on the use of old apple brandy and sweet potatoes, and generally with the happiest result. Often it is impossible to say in advance what will agree with our little patients. They often crave apparently objectionable articles of diet, but which on trial are found to agree admirably with them. Hence, we will often act wisely by consulting rather the suggestions of nature than the dictates of reason—remembering that there is much truth in the maxim, "*quod sapit, nutrit.*"

*Intestinal Hemorrhage in an Infant.*—The occurrence of hemorrhage from the bowels during infancy, though comparatively rare, is nevertheless sufficiently frequent to attract the notice of practitioners. Cases of it are recorded by many of the continental writers on diseases of children, and we have an account of one related by Dr. N. C. Stevens, in the *Boston Med. and Surg. Journal* for June 17, 1858. The patient was an infant three days old. Death promptly ensued. The following is an account of the appearances detected upon an examination of the body thirty hours after death.

"Rigor mortis very slight; the body—even its most depending portions—extremely white and bloodless. Brain not examined. On opening the chest and abdomen, all the organs appeared naturally developed. The heart and blood-vessels, the lungs, liver, pancreas, stomach, kidneys, and supra-renal capsules, were more or less pale, according to their structure, and entirely destitute of blood. The spleen, probably by reason of its structure at this age, seemed filled with dark grumous blood, which was easily pressed out on dividing the organ. The stomach contained a small quantity of partially digested milk. The entire alimentary canal was slit open, and its mucous surface exposed. The duodenum and upper portion of the jejunum were paler than usual; the lower portion of the latter, and the upper portion of the ileum exhibited various degrees of congestion, and were covered with dark grumous blood. Below this, and embracing about two feet of the middle of the ileum, it was of an ivory whiteness, and contained nothing but the natural mucous secretion, exhibiting a wonderful contrast to the portions above and below. The remaining portion of the ileum, and all the large intestines were congested, of a dark-red color, and covered with coagulated blood. The sub-mucous and peritoneal layers of the intestine, opposite the congested portions, were more or less discolored—probably a cadaveric change. On sponging the mucous surface carefully, no abrasion, softening, or other change was observable. The vessels usually observed traversing the intestines were empty; in fact, the body appeared to be completely drained of blood. The child was born of healthy parents. No hemorrhagic predisposition can be traced. No spot or pimple was observed during life. The remaining children, three in number, are healthy and robust."

*Remittent Fever of Children.—Etiology.*—In a paper on this disease by DR. J. LEWIS SMITH, contained in the *New York Journal of Medicine* for January, 1857, it is admitted that gastro-intestinal disease may, without doubt, produce fever of a remittent type in the impressible child, yet the following facts, derived from the author's observation of the disease, made exclusively in the upper portion of the island on which the City of New York is located, have convinced him that in a large majority of cases it is strictly of miasmatic origin.

"First.—The disease is very prevalent in spring and autumn, and rare in midsummer and midwinter, like other malarious affections. There are certain streets where he has known it to prevail almost like an epidemic, in the vernal and autumnal months. If the disease was, as Dr. Condie states, 'in every instance either a gastro-enteritis, an ileitis, or an entero-colitis,' how can this influence of the seasons be explained?"

We cannot perceive how, even admitting the etiology of the disease as suggested by Dr. Smith to be correct, that this would in any degree invalidate the views advanced by ourselves and others in respect to the leading pathological character of infantile remittent fever. That the disease may in many cases be generated by malaria we think there can be little doubt, but that it may be and is frequently developed independently of malaria, in the sense in which that term is employed by Dr. Smith, we are certain, from having met with it in numerous instances in localities where miasmatic diseases are unknown.

"Second.—Often, but not always, the remissions are more marked than would be likely to occur in a symptomatic fever. The child may appear almost well in the morning, but in the afternoon and evening exhibit such intensity of symptoms as to cause the greatest anxiety on the part of friends."

We are to recollect again, however, that it is a very common occurrence for the remissions in the fever under consideration to be so short and imperfect, as to approximate it very closely to those of a decidedly continued type. From the symptoms of the disease during life, and its usual progress in a vast number of cases, and likewise from the lesions so commonly discovered after death, it may be set down as a disease identical, in all essential characteristics, with the typhoid or enteric fever of the adult.

"Third.—The symptoms are not altogether such as we should expect to find in a purely local affection. The patient will sometimes, it is true, when asked where he feels pain, place his hand on the abdomen, and pressure upon the abdominal parietes not unfrequently produces great distress. Dr. Condie alludes to this tenderness, evidently believing it to be a symptom of inflammation, but I have always been satisfied that it was neuralgic, from the fact that pressure on the lumbar vertebræ, and frequently, also, on the chest and limbs, caused as much suffering as when it was made on the abdominal walls. The patient, if old enough, will complain, too, of aching of the head, back, and limbs, which is more the symptom of an independent fever than of inflammation. Again, constipation is ordinarily present, unless in the last stages of the disease. Intestinal irritation or inflammation sufficient to cause so intense and protracted a fever as is often present, would be more likely to cause diarrhœa."

But we would remind Dr. Smith that constipation is not a necessary nor invariable symptom of the remittent fever of infants even at the commencement of the attack. Very many cases are attended from an early period with frequent, small, thin, and offensive discharges from the bowels, attended sometimes with much straining and griping. The fever is almost always protracted, but it will seldom be found to merit the term intense, unless the latter be used in a sense different from that in which it is usually employed.

"Fourth.—Children, even nursing infants, take intermittent fever; why then may they not take remittent fever? In my class at the Dispensary children with these diseases are frequently brought in together.

"Fifth.—I have found that measures directed to the alimentary canal, beyond simple purgation, do more harm than good. They fail to ameliorate symptoms; they weaken and distress the child. Moreover, when remissions occur, quinine will materially abridge the disease."

Neither of these positions demands any particular comment. The first is not denied, and the second, even if sustained by the results of extended series of clinical observations made by different observers, would be insufficient to overturn the positive evidence furnished by the pathological anatomy of the true remittent fever of children.

"Sixth.—Death seldom occurs from this affection. In one or two fatal cases which have fallen under my observation, the result followed convulsions and coma; and Dr. Stewart remarks, 'Dissections have furnished but little light on the morbid condition of the system in remittent fever, for, on a fatal termination, the transmission to the brain is the usual course of the disease.' The mode of death, then, and the post-mortem appearances, do not comport with the doctrine that the intestines are the seat of the morbid process. Dr. Condie does not agree with Dr. Stewart, but attributes death to inflammation of the intestines. I do not think that the remittent fever which I have treated, if uncomplicated, ever terminates in this way, for I have never seen a case in which abdominal symptoms did not yield to simple means."

We have nowhere said that in every fatal case of infantile remittent fever, death was the immediate result of intestinal inflammation, while such is probably the case when death occurs in very acute cases, which very rarely happens; in most of the more protracted cases the patient, when they result fatally, perishes from the effects of mere exhaustion—the suspension of the digestive and nutritive functions, and the depraved state of the fluids thence resulting. To the negative statements made by Dr. Smith, we simply oppose the positive evidence as to the existence in the disease of intestinal lesions of an inflammatory character, presented by some of the most authoritative practitioners of Great Britain and continental Europe, to say nothing of that derived from our own limited post-mortem examinations in fatal cases of the disease.

"Seventh.—Continued fever of the adult is of rare, and infantile remittent of frequent occurrence in the locality of my practice. The latter is not then identical with the former, as it appears to be in London and Paris, from the descriptions given by Rilliet and Barthez, and West."

To this position we can only reply that our own experience, and the deductions from that experience, are directly the reverse of those of Dr. Smith.

The above facts appear to Dr. Smith to prove conclusively that the form of infantile remittent fever it has been his lot to treat, has been generally of a miasmatic character. He thinks it very important to understand the nature of the affection, as the treatment will vary according to the theory we adopt. If, living in a malarious region, we embrace the Broussain views of the American authors cited by him, and treat the fever as a local disease, we shall fail, he thinks, to ameliorate symptoms, and be mortified and discouraged by the result of our treatment, if he be permitted to judge from his own experience. His reliance, he informs us, is mainly on expectant measures until remissions occur, and then on the exhibition of quinine. In cases thus managed, convalescence, he remarks, has been more speedy and certain than when opium, calomel, and counter-irritation were employed to remove intestinal irritation or inflammation.

*Tracheotomy in Croup.*—This subject is very fully discussed by DR. THOMAS F. ROTCHESTER, in a paper contained in the *Buffalo Medical Journal* for July, 1858.

In view of a series of considerations set forth in the first part of the paper, he concludes that the question of tracheotomy in croup must necessarily give rise to the following interrogatories:—1st. Under what circumstances, if any, should it be performed, and by what complications is it absolutely prohibited? 2d. What subsequent measures may be regarded as accessory or necessary? 3d. Is the operation in itself or in its consequences hazardous to life?

*Circumstances that Warrant the Operation.*—"Adopting," he remarks, "the hypothesis of the 'croupous crasis,' if not absolutely, at least partially, must we, therefore, with M. Piorry and Jules Guerin, question the propriety of employing tracheotomy for the relief of a local manifestation of a general disorder? Their mode of reasoning might, with almost equal propriety, be urged against endeavoring to divert gout and rheumatism from the stomach or endocardium. Is the fact that the cavity of the trachea is almost invariably patent to a considerable degree a valid objection? It will be remembered that a membrane, if small, may, should one extremity become free, prevent egress or ingress of air, by valvular action. It will also be borne in mind that false membrane, acrid or inspissated mucus, or even an inflamed or turgid condition of the lining membrane of the larynx, unattended by secretion or exudation, may, unquestionably, give rise to long and repeated spasmodic occlusion of the glottis, a condition full of danger, which tracheotomy would render harmless. It next seems necessary to glance at the mortality of the disease. This, under various forms and combinations of treatment, Dr. Green's method possibly excepted, may be set down at two-thirds of all attacked; what the natural termination would be can only be a matter of inference; it is probable, however, that it would be fatal in nearly every instance; yet Dr. Ware tells us, that one of his patients, who was extremely ill, recovered spontaneously, he being so ungovernable as to successfully resist all attempts at medication." Dr. Rotchester also relates a case of unassisted recovery in a child two years of age. It had been ill several days—nothing was done, inasmuch as recovery was deemed impossible, the infant appearing to be moribund. The next day, however, it was better; inspection revealed a thick, false membrane on the tonsils. Recovery finally took place, but for three weeks the patient could not cry audibly, and his voice continued weak and hoarse for two months.

"Let us now look at the results of tracheotomy, and assume that three-fourths of those upon whom the operation is performed perish, although the statistics of Valleix make the proportion considerably less. We must remember that it has usually been employed as a last resource, when all other measures have failed, many of which, although proper in themselves, must to a certain extent prejudice the success of the operation. Tracheotomy should not be *contrasted* with the other means of treating croup, as being one mode in distinction from others, but it should be looked upon as a measure not to be neglected when all other rational treatment is evidently powerless; and in this aspect, to use the strong language of Mr. Henry Smith, of London, a success of but one in *ten*, or even one in *twenty*, justifies the procedure. The proper moment is not so much a matter of time, as of symptoms,—it may be within twenty-four hours of the seizure, or many days from the commencement of the attack. If, after general treatment, and especially topical treatment, has been fully and faithfully tried, the symptoms of suffocation are not relieved, or are become more threatening, and especially, if with persistent dyspnœa, there are manifestations of cyanosis, the operation should not be delayed. If, however, what may be termed the proper period has passed, may the trachea still be opened with any hope of permanent advantage? To this it may be replied, that if the symptoms are still chiefly referable to the upper portion of the air-passages, although conjoined with great exhaustion, the operation is advised by those who have practiced it most, even although from the previous failure of the vital force the patient may succumb to the shock produced by it. The age of the patient is an important consideration, the older he is, *cœteris paribus*, the greater the probability of a successful issue. Children under two years old 'invariably die,' says Trousseau, and such is the universal testimony. It is probable that the severity of the operation is too great for their tender age. It is possible that with them laryngotomy might, with advantage, be substituted for tracheotomy, as, comparatively, it is easily made and does not involve much time or loss of blood in its performance."

*Complications Prohibiting Tracheotomy.*—These Dr. Rotchester considers to be "rather those connected with the nervous and circulatory than with the pulmonary apparatus. They may be laid down as follows: delirium or stupor; excessive prostration; feeble and very frequent pulse; coldness and pallor, or great lividity of the surface. With reference to bronchitis and pneumonia there is a diversity of opinion. It is held by MM. Rilliet and Barthez, as also by M. Guersent, that the extension of the membrane to the smaller bronchi, or pneumonia of a single lung, are not to be regarded as insuperable objections, although they certainly diminish the chances of recovery. All persons who have seen much of membranous croup will admit the difficulty and often the impossibility of determining by physical exploration the exact condition of the lungs. There seems to be one valid argument against operating when either bronchitis or pneumonia are supposed to exist, viz.: the success that M. Trousseau claims to have accomplished since the adoption of the woolen cravat, which is used exclusively to prevent the supervention of pulmonary disease."

*Accessory Treatment.*—In respect to this may be mentioned, Dr. Rotchester remarks, a well-ventilated apartment, maintained at an equable temperature, ranging from 70° to 75°; the atmosphere being kept moist by the evaporation of warm water; the adaptation of a light, coarse woolen muffler about the throat, and enforced alimentation, if food is not taken readily. If from any cause respiration is suddenly suspended, whether immediately succeeding the operation or some time subsequently, the lungs should be carefully inflated through the artificial opening, or if the suspension is produced by a clogging of the trachea or larger bronchi, an attempt should be made to remove the obstacle by inserting a large flexible tube, open at both ends into the trachea, and drawing upon it quickly and forcibly with the mouth. Roux saved a patient by this expedient, removing a clot of blood with a female catheter.

Dr. Rotchester recommends the operation to be performed under the influence of chloroform or ether, which, while it robs this formidable operation of all its terrors, may, also, we are told, do away with much of its dangers.

*Danger of the Operation.*—To this, which is the subject of the third of Dr. Rotchester's interrogatories, he replies: "There can be but this response—the operation is *in itself* hazardous to life—hazardous from the shock, from exhausting hemorrhage, and from inspiration of blood. It is also, probably, more hazardous to life in its consequences than equally severe operations in other portions of the body. But admitting the full hazard, both of the operation and its consequences, the question recurs as to the comparative risk of the operation or of its neglect." To this Dr. Rotchester would respectfully submit, that a full, and he trusted impartial, investigation of the subject leads to the conclusion that while tracheotomy, from its intrinsic gravity, ought never to be lightly or precipitately resorted to, it affords a possibility of recovery when hope from other resources cannot reasonably be entertained, and that moment having arrived, it should not be neglected or delayed.

*Glycerine as a Local Application in Croup.*—DR. E. R. MAYER, of Wilksbarre, Pennsylvania, in the *American Journal of Medical Sciences*, April, 1858, calls the attention of the profession to the use of glycerine us a local application in croup, believing it a remedy from which much advantage may be derived. He refers to its use by him in two exceedingly severe and well-marked cases, that occurred within a few days of each other. The one in a boy eighteen months old, the other in a girl four years old. In both the disease was developed during the decline of the eruption of measles. Suffocation seemed imminent in both cases. The treatment consisted in prompt emesis with sulphate of copper, by which the severity of the symptoms was mitigated, and afterwards, in the rapid introduction of calomel into the system; the use of from one to three grains of sulphate of copper, dissolved in water, every two or three hours, alternated at the same intervals with tartar emetic in nauseating doses, or the still more effective sedative, the veratrum viride, in doses of from two to four drops of the fluid extract; small blister over the upper part of the sternum, and the application of pure glycerine to the glottis, whenever its use was indicated by a return or increase of the brassy, ringing cough, or crowing respiration. The application of the glycerine was in each instance soon followed by a manifest softening of the sound of the breathing and cough, and a considerable reduction of the dyspnœa and general distress. This improvement usually lasted for a few hours, but by applying the glycerine at short intervals Dr. Mayer found that he was able to afford the patient an amount of permanent relief throughout the disease. In the younger of his patients expectoration of softened false membrane in shreds and patches, "a genuine *detritus*, mixed with altered mucus and a little dark blood," took place twenty hours after the application of glycerine was commenced, and in the other patient on the fifth day of the attack. "The croupy symptoms did not entirely disappear in either case until a day or two after the expulsion of the false membranes, but the patients were soon convalescent. Expectoration was promoted and recovery hastened by the administration of small doses of quinine and of chlorate of potassa with syrup of senega."

Dr. Mayer applies the glycerine by pressing down and drawing forward the tongue with the finger, and squeezing the contents of a long, thick camel's-hair brush, dipped in the liquid, over, as near as possible, the chink of the glottis, and then swabbing the whole throat. A sponge or portion of charpie may no doubt be preferable in some cases to the brush.

*Belladonna with Mercurial Ointment as a Local Application in Croup.*—Dr. JOHN D. SHELTON, of Queen's County, New York, in the *American Journal of the Medical Sciences*, April, 1858, speaks in high terms of the application, by friction on the sound skin, over the trachea, in membranous croup, of an ointment composed of two drachms of the extract of belladonna combined with six drachms of the ung. hydrar. mite; the application being repeated every two hours. Two or three cases are related by him in which the ointment was employed in conjunction with other remedies, and apparently with good effects.

*Scarlet Fever; use of Quinine in.*—DR. EDWIN A. MORRISON, of Lawrenceville, Virginia, states, (*Virginia Medical Journal*, August, 1858,) that, in a practice of more than thirty-five years, he has repeatedly treated scarlet fever in its epidemic form. In 1837 it raged extensively in his section of the State. He treated the worst cases successfully with cold water affusions, as used by Dr. Currie, and he vainly believed that by this means he could control almost any case, but this opinion was soon shaken. In 1839 he encountered the epidemic again, when some of his cases defied the cold affusions and every remedy he employed. In 1856 he met it again. The first case to which he was called was that of a negro three years of age, when in a condition apparently hopeless. He ordered quinine, and directed the throat to be mopped with a solution of nitrate of silver. Contrary to his own expectation, and that of his friends, Drs. G. Mason and E. B. Jones, who saw the case frequently with him, the child recovered. A white female child, of delicate constitution, was the next patient seen by Dr. Morrison. The above-named physicians attended the case with him. No quinine was administered—the child died. Dr. Morrison cannot say positively how many cases he saw subsequently in this same family, but thinks about twenty. All of them were treated with quinine, and all but one recovered. The fatal case occurred in a negro boy about fifteen years old, whose condition was not made known until he was hopelessly ill. So soon as Dr. Morrison saw him he ordered quinine, but the lad survived but ten or twelve hours. Dr. Morrison treated ten or twelve cases in other families, all of which were put on the use of quinine, and all recovered. Early in May, 1858, the disease again appeared within the range of his practice; he treated about fifteen cases. One of these was a negro girl between ten and twelve years of age, who was speechless when first seen. She died in a few hours. Another was a negro child only two years old, who died of laryngitis after having survived the constitutional impression of the disease. The remaining cases, with two exceptions, have all fully recovered. The two excepted cases have had dropsical effusion. This day (June 5th) they are so much better, remarks Dr. Morrison, as to leave no doubt as to their speedy recovery.

The plan of treatment pursued by Dr. Morrison is to order quinine upon the occurrence of the first symptoms of the disease, in doses adapted to the age of the patient, repeated every two or three hours, until the system is brought under its influence. He gives, occasionally, a few grains of blue mass to move the bowels gently, and mops the throat with a strong solution of nitrate of silver. When the patient is old enough, he directs the use of a gargle of red pepper and common salt, or the throat to be washed with it. Dr. Morrison remarks that his friend Dr. R. Sims, of the same county, has employed quinine in some few cases of scarlet fever with the happiest effects.

*Yeast in Scarlet Fever.*—DR. A. S. MCCLEAN states, (*Boston Med. and Surg. Journal*, 1858,) that some years ago his attention was directed to the use of yeast in scarlet fever by an article from the pen of Dr. Smith, of Baltimore; he was in consequence induced to employ it during the winter of 1857–58 in fifty-three cases of the disease, being all that fell under his care during that epidemic. The whole of these cases recovered. The yeast was given from the onset of the disease in doses of from a tea to a table spoonful every two or three hours, and continued until the period of desquamation. In about two-thirds of the cases inunction with lard was employed in addition to the yeast.

*Acetic Acid in Scarlet Fever.*—DR. CASSELBERRY bears strong testimony in favor of the acetic acid as a remedy in scarlatina, (*Cincinnati Lancet and Observer*, February, 1858.) His mode of prescribing it is to combine two fluidrachms of the pure acid with three fluidounces of distilled water, and three fluidrachms of simple syrup, of which he gives as a dose a teaspoonful every three hours. At the same time he envelopes the patient in a sheet wrung out of tepid water, and continues it on from half an hour to an hour. He applies poultices of roasted onions to the lumbar region if there be a suspension of the urinary secretion. For the reduction of the glandular swelling of the neck he prefers the use of camphorated olive oil.

Diluted acetic acid is also spoken of in the highest terms, as a remedy in scarlet fever, by DR. B. F. SCHNECK, of Lebanon, Pennsylvania, (*American Journal of the Medical Sciences*, July, 1857.) Of sixty cases treated with the acid he states that he lost none. He prepares the system by an aperient of three to five grains of calomel, followed in two hours by castor oil, or a mixture of rhubarb and magnesia, applying at the same time around the throat, from ear to ear, a piece of flannel saturated with a mixture of soap liniment, f℥j; camphor liniment and laudanum, āā ʒij. After the bowels are opened he gives to a patient nine years old a tablespoonful of a mixture composed of from fʒj to fʒiv of the officinal distilled vinegar to f℥iv of water, sweetening it at the time it is given—the dose to be repeated every few hours, and the strength of the acid increased in proportion to the threatening nature of the symptoms. Dr. Schneck gives graduated doses of brandy two or three times a day, from the beginning of malignant cases, and on the second or third day in anginose cases.

*Veratrum Viride in Scarlet Fever.*—DR. J. FITHIAN, of Woodbury, New Jersey, states, in a communication contained in the *Medical and Surgical Reporter*, June, 1858, that in the winter and spring of 1857-58, he had employed in a number of cases of scarlatina Norwood's tincture of veratrum viride, much to his satisfaction. "I had not," he says, "known it to be used in scarlet fever, but it occurred to me that inasmuch as one of the most constant attendants of the disease is a quick, frequent pulse, it might control and bring down this rapid action, and thereby enable the system to bear up under the disease, promote the favorable action of other remedies, and leave it to run its course to a favorable termination. Upon trial I found this to be the case. Its effects were very decided, and while it will not—nor is it calculated to—supersede other remedies, it has in my hands thus far been one of the most efficient and satisfactory."

*Treatment of Scarlet Fever.*—In the same communication in which Dr. Fithian furnishes us with his experience of the effects of the veratrum viride in scarlatina, he gives a statement of his mode of treating the disease generally, which, as it comprises the therapeutic experience of a well-instructed and skillful physician, in one of the most unmanageable diseases of infancy, is invested with a special interest.

"Whenever," he says, "I am called to a case of scarlet fever, and the throat is at all sore, I use the cold cravat; that is, a napkin wrung out of cold water, or if there is much sensitiveness to cold, slightly tepid, extending from ear to ear, and from breast to chin, covered externally with a fold of dry flannel. If I do not, as is generally the case, see the patient until after the chilly stage has passed, in addition to the wet cravat I order calomel, in doses of from six to twenty grains, according to the age of the patient, followed in one hour by a suitable dose of castor oil. If the skin still continue hot, I use frequent ablutions of cool or cold water all over the body, with a little oil on the wet napkin used for the bathing. If the head is affected I lay upon it a wet towel, wet the hair thoroughly, and fold the towel over it, or use, at discretion, a bladder of ice. The object is to cool down the skin as near as possible to a natural heat, and keep it there, and prevent congestion of the brain where it is threatened. In addition to the oily napkin for bathing, I like a little salt grease twice a day—the skin of ham or salt pork is a very good article—to pass over the body; it allays the itching and soothes the patient, and with the cold affusion lessens the inflammation of the skin, which by the extent of the sympathies of the latter aggravates every other symptom. When the patient is old enough to gargle, I use the Cayenne pepper tea—an ordinary teaspoonful of pepper to a half pint of boiling water—and repeat it every three hours. Essence of Jamaica ginger of a proper strength is a good substitute. If there is ulceration of the throat, a solution of twenty grains of nitrate of silver to an ounce of water, applied twice a day by a brush or swab, will be found a valuable remedy.

"After the bowels are freely moved by the calomel and oil, it is seldom that any active catharsis will be found necessary during the progress of the disease. Castor oil, or a neutral mixture made by dissolving a Seidlitz powder in four or six large spoonfuls of water, with or without the addition of Rochelle salts—a tablespoonful given every hour until it operates—is as good a laxative as I have found, where there is a hot skin, and as readily taken. In the active stage of the disease the warm or tepid bath will be found a most useful remedy. When the patient is restless, the skin hot and highly colored, with hot extremities, a cool or tepid bath is best—when, however, the extremities are cool, the bath should be as warm as the patient can bear. From five to ten minutes is ordinarily long enough for immersion."

Dr. Fithian relates two cases to show the manner in which he administers the veratrum viride. He gives it in doses of one drop of the tincture every three hours, gradually increased, drop by drop, until its effects are produced, throughout the strictly febrile stage. He thinks that the occurrence of sickness and retching indicate that it has been carried sufficiently far. The heart then gives way—if it does not do so, the specific effect of the remedy will not be likely to ensue. When this specific effect is produced, it is better to diminish the dose of the veratrum a half drop or a drop, but to continue its use until the disease subsides. It generally acts, Dr. Fithian remarks, as a sudorific.

*Otorrhœa as a Sequela of Scarlatina.*—From a very instructive paper on this subject, with illustrative cases, by DR. L. TURNBULL, of Philadelphia, contained in the *Medical and Surgical Reporter* for April, 1858, we extract the following, which, if not in the exact words of the author, convey accurately his meaning.

Otorrhœa, resulting from scarlet fever, is a disease which, if it be allowed to become purulent and chronic, is very difficult to cure. Such is the statement of every one who has devoted much attention to the subject. In the practice of Dr. Turnbull, one case had existed for thirty-five years, another for twelve, and another, which, before it fell under his care, had been under treatment for seven years. In several other cases the duration was, respectively, one, two, and three years. The profession has not, at any time, been sufficiently alive to the great importance of so treating acute inflammation of the tympanum as to prevent, if possible, the destruction of the internal portion of the ear, and the subsequent otorrhœa. Were the same amount of care exercised in respect to the management of the diseased condition of the ear in scarlatina as of that of the throat, a much smaller number of children would become permanently deaf, or suffer from a destruction of the tympanum, or from chronic otorrhœa of months', or perhaps years' continuance.

*Treatment.*—In the early stage, when the scarlet fever is at its height, we must endeavor to arrest the acute inflammation of the ear by depletion, but care must be exercised, as this exanthem will frequently assume a low type. Here local depletion, by leeches and small cups, to the mastoid process and antitragus, should be employed as soon as acute pain is complained of. Sometimes the child may be too young to indicate the seat of pain, but expresses his suffering by sudden screams and by crying; pressure at the lower portion of the ear, however, will reveal the cause and seat of suffering. The local depletion should be repeated at intervals, and to such an extent as the strength of the patient will permit, and assisted by active purgation, with jalap, scammony, or senna tea, while, at the same time, we support the child's strength with nourishing diet, etc.

If the case is one that will not bear depletion, or we are called in too late, we must content ourselves with applying counter-irritation, and trying the effects of purgation. Should suppuration have commenced, indicated by a chill, with increased pain, of a darting and throbbing nature, with a sense of bursting in the ear, the meatus, on examination, being of a livid red, and the membrane of the tympanum red and swollen, the best plan is to give discharge to the purulent fluid collected within the tympanum by introducing a delicate cataract needle and puncturing that membrane. This will prevent it ulcerating its way out externally, causing a destruction of the internal ear, or even extending internally to the meninges of the brain, and cause not merely a destruction of the organ of hearing but of life also. Cases are on record in which the ulcerative process extended in such a direction as to open the carotid artery into the Eustachian tube, causing a fatal hemorrhage. The extension of the disease to the brain may give rise, also, to effusion or to disease of the membranes—death taking place from convulsions.

Ototis may likewise take on a sub-acute form, attended with a discharge of a muco-purulent or sero-purulent matter. In such cases the general treatment, which is of the greatest importance, should be directed to improve the condition of the blood by tonics—as iron, quinine, and cod-liver oil—with the frequent use of the bath, or wet towel, with frictions, and out-door exercise in clear weather. The local treatment consists in the application of solid nitrate of silver to the region of the Eustachian tube, every third day, with stimulating gargles, and the use of weak astringent washes for the internal ear, while most active counter-irritation is kept up by blisters, setons, or croton oil, applied over the mastoid region and tonsils. In some of the most unpromising cases the otorrhœa will gradually cease, and the disease may thus be cured; but if the case becomes chronic, then the treatment must be continued for months and even years.

*Belladonna as a Preventive in Scarlatina.*—DR. J. CHESTON MORRIS, of Philadelphia, relates, in the *American Journal of the Medical Sciences* for April, 1857, the result of a comparative trial made by him at the Foster Home, to test the powers of the belladonna as a prophylactic of scarlet fever.

The number of children subjected to experiment were forty-three; of these nineteen were placed under the influence of the belladonna, and twenty-four took none. Three grains ext. belladonnæ were dissolved in a mixture of half a fluidounce of distilled water, and one fluidrachm of alcohol. Of this solution one drop for each year of the child's age was given morning and evening. The effects produced were generally slight—a little dryness and redness of the fauces, and dilatation of the pupil, with occasionally a little headache, except in one case not included in the nineteen given above, a girl who had had scarlatina severely some years previously, to whom it was administered under an erroneous opinion that she had not had it. She had, from the action of the belladonna alone, a well-marked efflorescence, with slight fever, delirium, and sore-throat. Of those who took the belladonna, eleven had scarlatina regularly, and eight escaped. Of the twenty-four who took no belladonna, eighteen had scarlatina regularly, and six escaped. Of the eleven who sickened with scarlatina while using the belladonna, the sickening happened in two on the 6th day of its use, in two on the 8th, in one on the 10th, in one on the 15th, in two on the 17th, in one on the 40th, in one on the 41st, and in one on the 42d day.

"The last three cases were very mild; the others did not differ in any way from the cases occurring simultaneously in the other children. The last case among the twenty-four who took no belladonna occurred on January 12th, (sixteen days after the appearance of the disease,) while the belladonna cases occurred up to February 9th—forty-four days after the appearance of the disease. The sick children were carefully isolated, the others were mingled without reference to their taking or not taking the medicine, and were in all respects equally exposed. If we reduce the above figures to a per centage, for the sake of comparison, we find seventy-five per cent. of the unprotected were taken with the disease, while only fifty-three per cent. of those under the influence of the belladonna were affected. The difference in the period of incubation is also very striking." Dr. Morris thinks that this result is to be explained upon the supposition that the belladonna prevents, to some extent, the absorption of the scarlatinous miasm. "We know," he says, "that the process of absorption depends to a great extent on the movement of the blood in the blood-vessels—the slower this movement, and the fuller the blood-vessels, the less the absorption; hence the effect of narcotics would be to diminish absorption. Thus, too, the production of intoxication in cases of poisoned wounds prevents the absorption of the poison; and an old domestic prophylactic against marsh miasm was the taking a morning dram; we know also that absorption of miasmata takes place more readily when the stomach is empty than after a full meal. Why? Because, during digestion, the pulse becomes fuller and more frequent. If this explanation be the correct one, any other narcotic should produce quite as effectual a prophylaxis as belladonna. The next question is, should belladonna be given generally to all who are exposed to the influence of scarlatina, and who have not had the disease?" From the small numbers observed by him, Dr. Morris hesitates to answer definitely yes or no. "It is not," he observes, "such a trifle as it has been represented, to maintain even a slight narcotic impression for a month or six weeks, and at every fresh exposure the patient must go through a fresh course of the medicine. Yet where an epidemic is very malignant, or where a hereditary fatality attends the disease in a family, I should recommend the administration as tending to diminish the risk of contracting the disease."

*Vaccination.*—The following are a portion of the able remarks on the subject of vaccination generally, contained in a report presented by DR. GORDON W. RUSSELL, of Hartford, to the Connecticut Medical Society, at its session for May, 1857, (*Proceedings of the Sixty-fourth Annual Convention of the Connecticut Medical Society.*)

"The propriety of using the scab or crust for vaccination, instead of the lymph taken about the sixth or seventh day, may well be doubted; not but that a good crust, consisting of hardened lymph, will equally communicate the disease, but the difficulties and uncertainty of obtaining a crust consisting of lymph alone, becomes an objection. Frequently the vesicle is broken and the lymph nearly discharged, or in consequence of inflammation, the crust consists in part of dried purulent or semi-purulent matter, which may produce a sore, but not always a vaccine vesicle, and which, if not carefully examined on the fifth day may be thought to be a genuine one. And here I may be allowed to suggest the propriety of testing all cases of primary vaccination, this would afford a sure and perfect guaranty of the protection given by the first. It is attended, I admit, with some trouble, but if the fee is not already remunerative it should be made so. That revaccination is a necessary operation, protecting the system, already wholly or partially exposed to variolous infection, is, I believe, generally admitted, though still denied by some physicians. I confess I have rarely seen a perfect vesicle in a secondary vaccination, when there was already a well defined, punctated scar from the first. I have occasionally seen near approaches to it, varying, however, in the constitutional symptoms, in the appearance of the areola, and of the attendant inflammation. Some of the profession, I know, are reported to have seen it frequently, but I have not been so unfortunate, the nearest approach to it that I have seen this season was in a lady of at least *sixty* years of age, who had been inoculated with variolous matter in Scotland when a child. Of course the crust that is formed during these revaccinations, should never be used for the purpose of producing the vaccine disease. It might occasionally answer, and, of course, it would do so whenever it is the product of a genuine vesicle, but such is so rarely the case that it is safe to lay it down as a general rule that it should never be used.

"There is an interesting portion of this subject, itself alone sufficient for a lengthy dissertation, viz.: upon *the communicability of disease by vaccination;* this demands careful and thorough investigation, both to satisfy ourselves, and quiet the public feeling, which is strongly inclined to believe in its truth. Without denying the possibility of it, I am still somewhat skeptical as to its frequent occurrence, though cutaneous disease may occur after its performance and be the indirect result of it, as vaccination often developes any tendency to disease of the skin which may be present in the system. I can now recall but one instance in which I have witnessed any cutaneous eruption to follow vaccination this season; this was eczematous in its character, and appeared about the tenth or twelfth day of the operation, behind the ears and upon the lower part of the scalp. The child was teething at the time, which was enough, in my opinion, to account for it, and that it was owing to this and not to the vaccine infection, was shown by the same matter being used in quite a number of instances, both before and afterwards, without any such result being produced. If physicians would make a careful record of all such cases that come under their notice, a mass of information would be obtained, which would enable us to dispel many of the illusions of the public.

"The popular notion that vaccination should be repeated at every certain and defined period of one's life is altogether wrong; there can, with reason, be no such course marked out. If, however, the operation was done during infancy, it is proper that it should be tested after the subject has grown up. If anything further than this is done, it must be as much to satisfy the wishes and quiet the fears of the timid, as to comply with any rule indicated by reason or experience."

